# Lower extremity joints and their contributions to whole limb extension

**DOI:** 10.1080/23335432.2019.1695540

**Published:** 2019-12-04

**Authors:** J. W. Fox, A. E. Jagodinsky, C. M. Wilburn, L. Smallwood, W. H. Weimar

**Affiliations:** Kinesiology, Auburn University, Auburn, AL, USA

**Keywords:** Isometric, kinetics, strength

## Abstract

Lower extremity multi-joint strength curves tend not to evaluate individual joint contributions to endpoint force in maximum effort isometric whole limb extension. Therefore, the purpose of this study was to measure the contribution of the hip, knee, and ankle to vertical ground reaction force in maximum effort isometric whole limb extension at various postures. An effect of posture on the contributions of the hip, knee, and ankle to vertical ground reaction force was found (*F_(3,96)_* = 85.31, *p* < 0.0001; *F_(3,96)_* = 21.32, *p* < 0.0001; *F_(3,96)_* = 130.61, *p* < 0.0001 for the hip, knee, and ankle, respectively). The hip and knee contributed most to vertical endpoint force when the lower limb was in a flexed posture, and their contributions decreased when posture was extended. Conversely, the ankle contributed least when the limb was flexed, but its contribution increased as posture was changed from flexed to more extended. In comparison to recent research involving induced acceleration analysis, it appears that the hip, knee, and ankle utilize the same force allocation strategy in multi-joint maximum effort isometric leg extensions and activities of daily living.

## Introduction

Typically strength has been measured via single-joint strength curves for various joints throughout the body (Kulig et al. 1984; Bober et al. 2002; Anderson et al. 2007). Single-joint strength curves are useful due to the connection between muscle tension and muscle length (Gordon et al. 1966), and muscle length and joint angle (Shadmehr and Wise 2005). As a result, single joint strength curves take on one of the three different shapes: ascending, descending, or ascending-descending (Kulig et al. 1984). To some degree, the characteristic shapes of single-joint strength curves reflect the portion of the length-tension curve over which the muscle or muscle group operates (Kulig et al. 1984; Anderson et al. 2007). Therefore, single-joint strength curves might provide a means for optimizing musculoskeletal model output (DeSmitt and Domire 2016).

Despite the importance of single-joint strength curves, many activities of daily living are multi-joint efforts. Hugh-Jones (1947) measured multi-joint leg extension capacity and found that endpoint force increases as leg extension increases. Similar results have been found in research aimed at athletic populations (Papadopoulos et al. 2008; Hahn 2011), as well as in workplace environments (Rees and Graham 1952; Pheasant et al. 1982; Lee 2007). Hahn (2011) estimated joint torques during maximum effort isometric leg extensions. Strong correlations were found between joint torques and endpoint force when the knee was flexed 60 degrees or more, indicating stronger associations between joint torques and endpoint force when force capacity is decreased (Hahn 2011). While statistical perspectives on joint contributions to lower extremity endpoint force are useful, a clear mechanical description of joint contributions to endpoint force across postures is lacking.

Clinical research has also utilized statistical models to understand the relationship between multi-joint force capacity and functional tasks. Endpoint force in a lower extremity multi-joint effort correlates moderately with functional tasks such as gait or sit to stand (Azegami Pääsuke et al. 2004; Masako et al. 2007). Contribution of individual joints to activities of daily living was examined by Hasegawa et al. (2008) by utilizing lower extremity single-joint strength measures to statistically discriminate between functionally independent and dependent groups of elderly adults. Discriminant analysis indicated that hip extensor strength is most important when performing functional tasks (Hasegawa et al. 2008). While statistical models of the relationship between joint capacity and limb capacity are valuable, they do not provide a physical understanding of joint contributions to lower limb force capacity.

Although research into multi-joint isometric force capacity is lacking in its description of individual joint contributions, a body of research has grown that has examined the contribution of individual joints and/or muscles to center of mass acceleration. For example, induced acceleration analyses examined the contribution of lower extremity joints and/or muscles to acceleration in walking (Kepple et al. 1997; Hof and Otten 2005; Kaya et al. 2006; van Antwerp et al. 2007), running (Hamner and Delp 2013), sit-to-stand (Caruthers et al. 2016), and vertical jump (Suzuki et al. 2018). These studies demonstrate the role of individual joints within various multi-joint movements. However, the knowledge provided by induced acceleration analyses lacks a strength curve reference for evaluating the relative efforts of joints involved in multi-joint movements.

In essence, a multi-joint lower extremity strength curve with a description of the contribution of individual joints to endpoint force is needed, because current multi-joint strength research does not provide a mechanical description of the contributions of individual joints to endpoint force, and a generalizable comparison for joint contribution within a multi-joint movement is lacking. Physical descriptions of the contribution of individual joints within a multi-joint isometric effort may provide insight into strategies utilized in various multi-joint movements. Such information could help explain biases toward particular joints in endpoint force contribution across involved joints, and thereby inform exercise and training, ergonomic design, and rehabilitation. Therefore, the purpose of this study is to quantify the force contributed by the whole lower limb, hip, knee, and ankle to vertical ground reaction force while performing a multi-joint maximum effort isometric leg extension task. Because the thigh and shank approach the singular position when the lower limb is extended, it was hypothesized that the contributions of the whole limb and ankle to vertical ground reaction force would increase while the contributions of the hip and knee would decrease as the leg is moved from a flexed posture to an extended posture, similar to postures recorded in vertical jump.

## Methods

Participants between the ages of 19 and 35 were recruited for participation in this study. Sixteen male and 17 female participants volunteered (height, 1.73 ± 0.09 m; mass, 76.9 ± 14.2 kg). Participants self-reported engaging in resistance exercise twice weekly. Also, participants self-reported no injuries within the past 6 months, and none reported any pain throughout the study. This study was approved by the Institutional Review Board at Auburn University (Protocol Number: 14–219 EP 1406), and all participants signed the informed consent.

Vertical jump was selected as movement for standardizing postures in the multi-joint maximum effort isometric leg extension protocol. Vertical jump was selected due to its multi-joint nature and due to the requirement to direct most of the energy in the vertical direction when attempting to jump to a maximum height (Zajac et al. 1984). Participants were asked to perform three maximum height vertical jumps. The jump in which the center of mass was elevated to the highest height was used to determine postures for the maximum effort isometric leg extension protocol.

**Figure 1.**
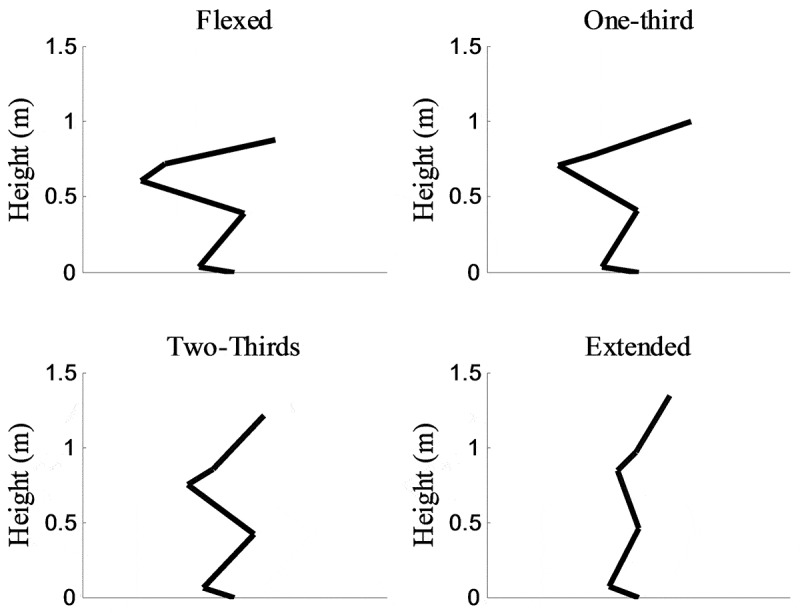
Example postures from a single participant. An immovable bar was fixed across the shoulders. Each foot was placed on a force platform. Participants were placed in a posture and asked to push vertically with as much force as possible

Postures used in the multi-joint maximum effort isometric leg extension were standardized based on the kinematics of each participant’s maximum height vertical jump. The range of motion utilized in the vertical jump was reduced to four postures: flexed, one-third, two-thirds, and extended (Figure 1). The flexed posture was obtained from the position of maximum knee flexion in the vertical jump. The extended posture was limited to 160-degree knee angle, as this is the limiting knee angle for endpoint force (Hugh-Jones 1947). The remaining postures were at one-third and two-thirds of the knee angle range between the extreme knee angles. For each of these postures the vertical locations of the acromion, greater trochanter, lateral tibiofemoral joint, and lateral malleolus were kept to ensure participants attained the correct postural configuration.

The multi-joint maximum effort isometric leg extension protocol was limited to four postures for two reasons: 1) to avoid fatigue of participants, and 2) because previous studies have demonstrated a curved relation between endpoint force and knee angle (Hugh-Jones 1947; Papadopoulos et al. 2008; Hahn 2011), this is enough data points to recognize a curved trend (Macon and Spitzbart 1958). From each of the four postures participants performed two maximum effort isometric leg extensions against an immovable bar for a total of eight trials. A squat rack was situated over two force platforms. Participants were asked to push vertically against an Olympic lifting bar with as much force as possible, since most of the energy in vertical jump is directed vertically. Squat rack safety bars were utilized as mechanical stops to prevent the bar from moving. The bar was set to the height of the acromion so that participants could push against the bar with it positioned across the shoulders. Participants were asked to posture themselves such that the heights of the greater trochanter, lateral midline of the knee, and the lateral malleolus matched the heights measured at the selected postures from the vertical jump protocol. Height of the anatomical landmarks was measured with tape measure prior to initiation of the trial to ensure the desired posture. The trial in which the participants demonstrated the greatest vertical endpoint reaction force at each posture was utilized for analysis.

The unit vector means and standard deviations for the vertical component of force were near unity (0.981 ± 0.008, 0.986 ± 0.007, 0.991 ± 0.004, and 0.993 ± 0.004, for the flexed through the extended configurations, respectively). This demonstrates that participants were able to follow instructions and pushed nearly vertically. As such the focus of this evaluation is on the vertical component of the endpoint force.

For each protocol participants donned compression clothing to minimize motion of retroreflective markers. Markers were placed on the calcaneus, head of the first metatarsal, head of the fifth metatarsal, medial malleolus, lateral malleolus, medial joint line of the tibiofemoral joint, lateral joint line of the tibiofemoral joint, and greater trochanter for each leg. Markers were placed on the pelvis at the ASIS, PSIS, and iliac crests for both right and left sides. Markers were placed on the trunk at the right and left acromion process and at C7. In addition to markers placed at specific anatomical landmarks, clusters of four markers were placed on the trunk, pelvis, right and left thighs, and right and left shanks to assist in tracking the location and orientation of each segment. Ten Vicon T-series cameras (Vicon, Los Angeles, CA, USA) were used to measure marker positions with a sampling frequency of 200 Hz. Two AMTI OR6-1000 force platforms (Advanced Mechanical Technology, Inc., Watertown, MA) were used to evaluate ground reaction forces at a sampling frequency of 1000 Hz.

Marker position data were exported to Visual 3D (C-Motion, Bethesda, Maryland, USA) for computation of center of mass location during the vertical jump protocol. Segment inertial parameters and segment center of mass positions were estimated based on previous standards (Hanavan 1964). The center of mass was computed for each frame of the vertical jumps. The jump in which the greatest center of mass height was achieved was kept for identifying postures of interest.

Kinematic and kinetic data were exported to Visual 3D (C-Motion, Bethesda, Maryland, USA) for computation of joint angles and joint torques. Joint angles were computed using an X, Y, Z rotation sequence. Hip angles are defined by the orientation of the thigh in the global frame, and a positive rotation indicates extension, adduction, and internal rotation (Note: Participants were facing in the negative y-direction). Knee and ankle angles were defined by the orientation of the distal segment in the frame of the proximal segment. Extension and dorsiflexion are positive rotations for the knee and ankle, respectively. Hence, the lower limb was considered to have 5 degrees of freedom. The joint angles for the hip, knee and ankle at the time of peak force were used for obtaining the Jacobian chain configuration.

Inverse dynamics was used to compute joint moments at the hip, knee, and ankle. Joint moments at the time of peak force were obtained and exported along with joint angles to MATLAB (MathWorks Inc., Natick, MA, USA) to analyze the endpoint force contribution from each joint. The Jacobian can be utilized to compute endpoint force given that the joint torques are known as illustrated below (Yoshikawa 1990; Valero-Cuevas 2016; Hagen and Valero-Cuevas 2017):

(1) $$\vec{F}=J^{-T}\tau$$

where *F* is the endpoint force vector, *J^−T^* is the inverse transpose of the Jacobian, and *τ* is the vector of joint torques. Hence, the equation was used to compute the contribution of each joint torque to the overall endpoint force. This was done for right leg at each of the four postures.

Force traces from the right leg in each postural configuration were examined for peak vertical force. This within-trial maximum was normalized to the between-trial maximum of the four conditions to demonstrate the posture of greatest apparent strength. Similarly, the vertical endpoint force attributable to each degree of freedom for each posture was normalized to the between-trial endpoint force maximum. In this way, all vertical force values are normalized to the maximum vertical force capacity of the leg, which highlights the joint contribution relative to whole limb capacity.

The normalized peak vertical endpoint forces and the normalized forces due to each degree of freedom were examined for an effect of posture using six separate one-way repeated measures ANOVAs. Tukey’s HSD was used to make pairwise comparisons between postural conditions for each dependent variable. The alpha level was set *a priori* at 0.05.

## Results

**Figure 2.**
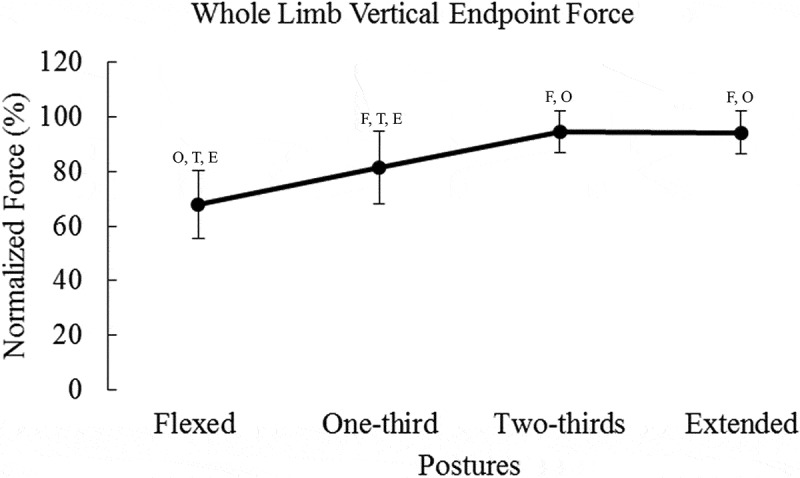
Vertical endpoint force increased as posture changes from flexion to extension. Statistical significance is denoted by the letters F, O, T, and E, which represent Flexed, One-third, Two-thirds, and Extended postures, respectively. The peak force in the trial was normalized to the overall maximum for each participant

**Figure 3.**
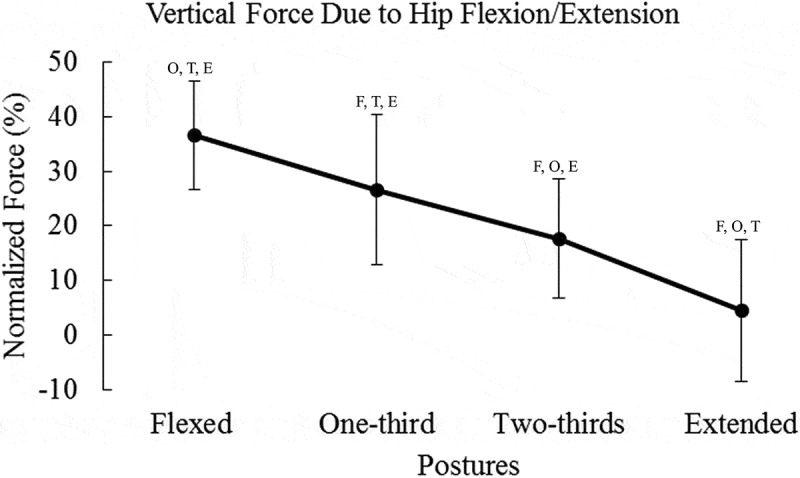
Vertical endpoint force due to the hip flexion/extension degree of freedom decreased as posture changed from flexion to extension. Statistical significance is denoted by the letters F, O, T, and E, which represent Flexed, One-third, Two-thirds, and Extended postures, respectively. Force due to the hip was normalized to the peak force in the postural condition

**Figure 4.**
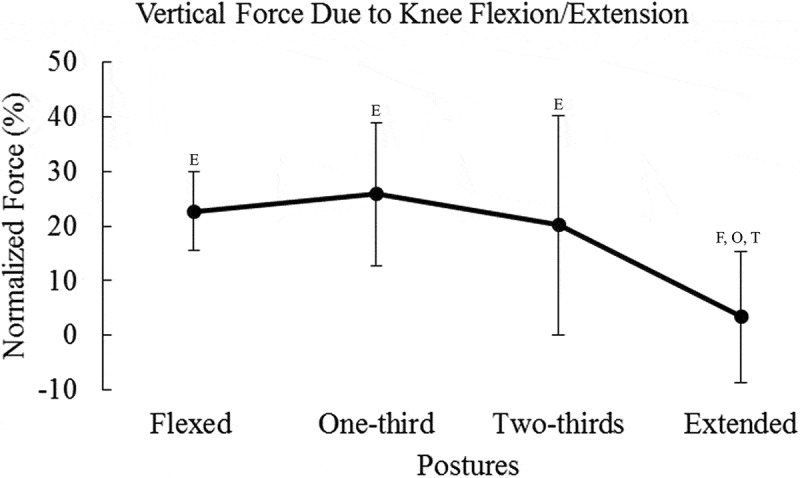
Vertical endpoint force due to the knee flexion/extension degree of freedom decreased as posture changed from flexion to extension. Statistical significance is denoted by the letters F, O, T, and E, which represent Flexed, One-third, Two-thirds, and Extended postures, respectively. Force due to the knee was normalized to the peak force in the postural condition

**Figure 5.**
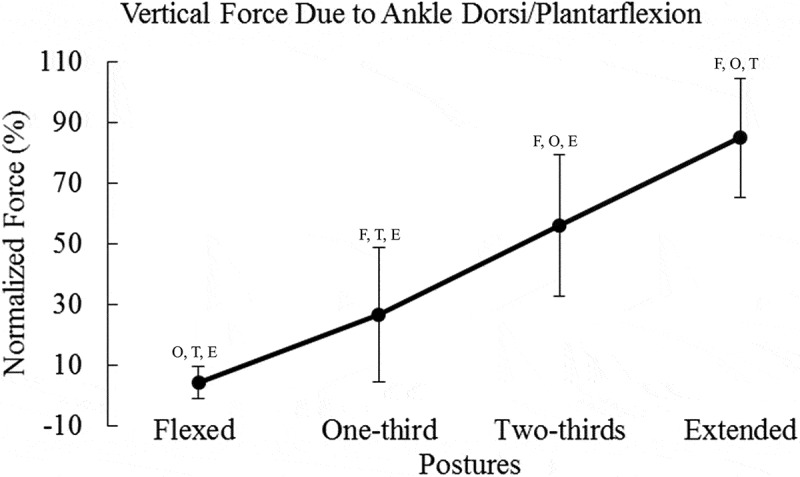
Vertical endpoint force due to the ankle dorsiflexion/plantarflexion degree of freedom inreased as posture changed from flexion to extension. Statistical significance is denoted by the letters F, O, T, and E, which represent Flexed, One-third, Two-thirds, and Extended postures, respectively. Force due to the ankle was normalized to the peak force in the postural condition

Means and standard deviations are presented in Table 1. Results for peak vertical force can be seen in Figure 2. Contributions of the hip, knee, and ankle to vertical endpoint force can be seen in Figures 3–5, respectively.

**Table 1.** Percent vertical force due to each degree of freedom, X and σ represent mean and SD

	Flexed		One-third		Two-thirds		Extended	
	X (%)	σ (%)	X (%)	σ (%)	X (%)	σ (%)	X (%)	σ (%)
Hip flexion/extension	36.60^O, T, E^	9.91	26.63 ^F, T, E^	13.69	17.61 ^F, O, E^	10.93	4.48 ^F, O, T^	13.02
Hip adduction/abduction	0.80	1.20	0.65	1.29	0.35	1.87	1.00	2.20
Hip int./external rotation	1.40 ^O, T, E^	0.91	0.61 ^F, T, E^	0.60	0.22 ^F, O^	0.16	0.04 ^F, O^	0.08
Knee flexion/extension	22.73 ^E^	7.19	25.90 ^E^	13.15	20.17 ^E^	20.10	3.38 ^F, O, T^	12.02
Ankle dorsi/plantarflexion	4.12 ^O, T, E^	5.32	26.45 ^F, T, E^	22.18	56.18 ^F, O, E^	23.39	84.99 ^F, O, T^	19.65

F, T, O, and E represent significant differences compared to flexed, one-third, two-thirds, and extended postures, respectively. Values are percent contribution to peak vertical force.

When comparing vertical force, there was an effect for postural condition (*F_(3,96)_* = 57.22, *p* < 0.0001, *η*^2^ = 0.983) (Figure 2). The greatest vertical force was produced in the two-thirds posture followed by extended, one-third, and flexed postures, respectively. There was no significant difference between the two-thirds posture and the extended posture, but all other pairwise comparisons were statistically significant (*p* < 0.0001).

An effect for posture on the contribution of hip flexion/extension torque to vertical force was found (*F_(3,96)_* = 85.31, *p* < 0.0001, *η*^2^ = 0.988) (Figure 3). Pairwise comparisons demonstrated that all postural conditions were significantly different in vertical force contribution (*p* < 0.0001). Therefore, as posture changed from flexed to extended, the hip flexion/extension contribution to vertical force decreased.

Changing leg posture from flexed to extended had no effect on vertical force contribution from the hip adduction/abduction degree of freedom (*F_(3,96)_* = 0.92, *p* = 0.4361, *η*^2^ = 0.478).

However, postural changes had an effect on the contribution hip internal/external rotation degree of freedom to vertical force production (*F_(3,96)_*= 46.51, *p* < 0.0001, *η*^2^ = 0.980). Pairwise comparisons showed significant differences for all comparisons (*p* < 0.0001), except between the two-thirds and extended postures. Hence, vertical force attributable to hip internal/external rotation tended to decrease as posture moved from flexed to extended.

Altering posture had an effect on the contribution of the knee flexion/extension degree of freedom to vertical force (*F_(3,96)_* = 21.32, *p* < 0.0001, *η*^2^ = 0.955) (Figure 4). The contribution of knee flexion/extension was significantly greater in the flexed, one-third, and two-thirds postures compared to the extended posture (*p* < 0.0001). The mean contribution of the knee tended to decrease with extension of the limb, and vertical endpoint force was significantly less compared to the other postures.

In contrast to the hip and knee, the ankle contribution to vertical endpoint force followed an upward trend (Figure 5). Changing limb posture had an effect on the contribution of the ankle dorsiflexion/plantarflexion degree of freedom to vertical force (*F_(3,96)_* = 130.61, *p* < 0.0001, *η*^2^ = 0.992). Pairwise comparisons showed statistically significant differences between all postural conditions (*p* < 0.0001). Therefore, ankle dorsiflexion/plantarflexion provided more vertical endpoint force as the posture changed from flexion to extension.

## Discussion

This study evaluated the percent contribution of the hip, knee and ankle to vertical ground reaction force while performing a multi-joint maximum effort isometric leg extension. The results of this study confirmed the hypotheses that the contributions from the hip and knee would decrease as the leg posture was changed from flexed to extended, whereas the ankle contribution would increase. Comparisons between these results and the literature provide an understanding of force allocation across joints in multi-joint movements.

Similar to previous literature, the results of this study show that as one’s legs become more extended the ability to apply force to an external object increases (Dallmann, Dürr, & Schmitz, 2016; Hugh-Jones 1947; Hahn 2011). However, this study reports the vertical component of the force as opposed to the resultant force. Control of the direction of force was attempted by providing instruction to push vertically. As reported above, participants were able to execute vertical pushes well. Such control over the direction of force is important experimentally. For instance, a change in direction of force will result from a change in the distribution of joint moments (Gruben and López-Ortiz 2000; Hof 2001). Therefore, the ability of the participants to push vertically provided a means to gain insight into how the hip, knee, and ankle contribution to vertical force changes for various postural configurations. In essence, this study demonstrates that posture is an important element in determining how joint torques are transformed into the vertical component of force.

Induced ground reaction force analyses (Kaya et al. 2006; van Antwerp et al. 2007) and induced acceleration analyses (Kepple et al. 1997; Hof and Otten 2005; Hamner and Delp 2013; Gu et al. 2015; Caruthers et al. 2016; Suzuki et al. 2018) employ such transformations to parse out the contributions of muscles to activity. For example, induced acceleration analysis has shown that the ankle moment contributions to vertical acceleration of the center of mass during vertical jump is greater than the contributions of the hip and knee (Suzuki et al. 2018). Contributions of the hip and knee to vertical acceleration occurred when the posture was more flexed, and this contribution decreased as the limb extended. Conversely, the ankle contributed when the limb was more extended. Similarly in this study, the ankle contributed a larger percentage of whole limb endpoint force capacity compared to the hip and knee. Furthermore, while the hip and knee contributions were largest in the flexed and one-third posture, the ankle contribution was greatest in the two-thirds and extended postures. Therefore, the joint contributions estimated in this study mimic the trends of joint contributions in vertical jump, which shows that multi-joint maximum effort isometric leg extensions can provide practitioners with a valuable reference for training for sport.

Joint contributions in this study also relate to joint contribution strategies in gait. For instance, the ankle contribution to vertical acceleration of the head, arms and, trunk exceeds 90% during single limb support phase of gait, whereas the knee and hip contribute little during this phase (Kepple et al. 1997). Likewise, estimates of work and power during walking show that the ankle produces more work and power during single limb support compared to the hip and knee across a range of gait speeds (Jin and Hahn 2018). The current study shows that when the limb is in an extended posture, similar to postures seen in single limb support phase of walking, the ankle is capable of providing the greatest contribution to vertical force. In essence, the structure of the lower limb is such that when it is extended, ankle moments transform into large vertical endpoint forces, which induce high magnitude vertical accelerations and substantial positive work during gait. In contrast, the lower limb is structured such that hip and knee moments transform into low magnitude vertical force, when the limb is extended. However, postures can be attained that shift the major contributions from the ankle to the hip and knee. For example, Caruthers et al. (2016) divided the sit-to-stand task into three phases and found that the gluteus maximus, followed by the vastus lateralis, provided the largest contribution to vertical acceleration in the early phases, which exhibit postures of greatest flexion. Additionally, the soleus provided the greatest contribution to vertical acceleration for the whole movement, although its contribution occurred in the late phase, which exhibit extended postures. The present study shows a similar result. In flexed postures the hip and knee provided the greatest vertical force, whereas in the extended posture the ankle made the larger contribution. In essence, the limb is structured such that the hip and knee moments transform into large vertical force while the limb is in a flexed posture, but the force allocation shifts to the ankle as the limb becomes more extended. The similarities in the results of these studies and the present study demonstrate the strong influence of limb structure and posture on force allocation strategies in multi-joint movements. Therefore, maximum effort multi-joint isometric tasks may provide valuable information to the clinician for identifying the degree of functional limitation and ensuing care for restoration of limb capacity.

However, there are limitations to establishing maximum effort multi-joint isometric tasks. For example, a control variable to explain force capacity is elusive, which makes development of a multi-joint strength curve difficult. Muscle force and joint torque are explained by kinematic variables such as length (Gordon et al. 1966) and joint angle (Kulig et al. 1984; Bober et al. 2002), respectively. Previously knee angle has been used as the control variable for multi-joint isometric leg extensions (Hugh-Jones 1947; Papadopoulos et al. 2008; Hahn 2011). Knee angle is a valuable kinematic variable for describing force capacity of the limb as long as the lower limb can be modeled as a 2 degree of freedom mechanism (Yoshikawa 1990). In the present study postures from each participant’s vertical jump were used as a control, and this involved more than two joints. Hence, the Jacobian was the kinematic variable, but it is not single-valued and unwieldy compared to knee angle. Therefore, identifying the most appropriate kinematic variable to explain multi-joint force capacity is an important topic for future research in this area.

Despite this limitation, the present study examined the relative contribution of each joint to maximum effort multi-joint isometric leg extensions. In doing so the results demonstrate that joints utilize a similar strategy for vertical force allocation seen in vertical jump, walking, running, and sit-to-stand, which is posture dependent. It is possible that strategies for force allocation in everyday movements occur in proportion to maximum effort isometric tasks. If this is true, knowledge of such proportions may provide practitioners with a means for evaluating functional limitations and developing appropriate training responses. Therefore, an appropriate next step is to construct single-joint force contribution curves from multi-joint efforts, which may provide valuable estimates of an individual’s capacity to perform athletic activities and activities of daily living.
